# Dynamic graph vector autoregression for characterizing cardiac electromechanical dynamics during incremental exercise from the joint phase space of electrocardiogram and phonocardiogram

**DOI:** 10.3389/fphys.2026.1946312

**Published:** 2026-09-29

**Authors:** Xiaohong Ye, Qiang Lu, Qianyu Lin

**Affiliations:** 1 Chengyi College, Jimei University, Xiamen, China; 2 School of Science, Jimei University, Xiamen, China

**Keywords:** cardiac electromechanical coupling, dynamic graph, electrocardiogram, incremental exercise, phonocardiogram, vector autoregression

## Abstract

Abnormal cardiac electromechanical coupling (cEMC) is associated with exercise-induced arrhythmias, yet monitoring cEMC during unrestricted movement remains challenging. While electrocardiogram (ECG) and phonocardiogram (PCG) recordings provide indirect measures of cardiac electrical and mechanical activity, each represents only a one-dimensional scalar observation of the cardiac system, and the system’s latent state-space dynamics response to exercise intensity remains inaccessible. Therefore, this study employs Takens’ delayed embedding phase-space reconstruction method to reconstruct the latent state trajectories and then combines them into a joint ECG-PCG phase space. Building on this representation, this study proposes dynamic graph vector autoregression (DG-VAR), a data-driven analytical approach for characterizing latent cEMC from the joint phase space of ECG and PCG during exercise. Benchmarking with an open-access database demonstrated that DG-VAR achieved the highest performance in predicting the joint phase space of ECG and PCG during exercise. The method yielded an average root mean square error (RMSE) of 0.0186 and mean absolute percentage error (MAPE) of 7.76 for ECG phase space predictions, and an average RMSE of 0.0085 and MAPE of 4.45 for PCG phase space predictions. Furthermore, matrix-derived parameters, including stationarity, coupling strength, and dynamic graph topology, showed significant correlations with cardiac electromechanical intervals. Analysis of these parameters revealed an intensity-dependent regulatory pattern in cardiac electromechanical dynamics: forward electromechanical coupling increases monotonically, the feedback mechanism rises initially and then stabilizes, and the network dynamics topology evolves towards a state of greater density and smoothness. This non-invasive, label-free, data-driven analytical framework offers a novel, physiologically interpretable approach to dynamic monitoring of exercise-induced cEMC responses.

## 1 Introduction

Exercise-induced arrhythmias represent a significant concern in sports medicine and exercise physiology, especially among individuals participating in competitive sports or recreational training (Harris et al., 2021; Polyák et al., 2023). Evidence suggests that these arrhythmias may result from transient destabilization of cardiac electromechanical coupling (cEMC) during exercise stress (Charisopoulou et al., 2019). Given that exercise-induced cardiac risk manifests during physical exercise, further investigation is required to develop monitoring approaches for cEMC dynamics during exercise.

Echocardiography serves as the clinical standard for evaluating myocardial mechanical function, offering functional indicators such as ventricular wall motion, ejection fraction, and strain. When combined with electrocardiography (ECG), it enables assessment of cEMC parameters, including the time to peak during the systolic phase (Lang et al., 2015; D’Ascenzi et al., 2020). However, echocardiography requires the probe to be held firmly against the chest wall, which complicates data acquisition and limits its application for studying cEMC during exercise (Sawada and Thomaides, 2009). In contrast, the phonocardiogram (PCG) offers a noninvasive marker of mechanical contraction by capturing low-frequency acoustic signals generated by valvular closure and myocardial vibration (Springer et al., 2016). The first (S1) and second (S2) heart sounds indicate key mechanical events: the onset of ventricular contraction and the closure of the aortic valve, respectively. Therefore, combining ECG and PCG enables inference of cardiac electromechanical intervals, including Q-S1, Q-S2, and S1-S2 intervals (Guo et al., 2022; Wang et al., 2022). Multimodal fusion of synchronously recorded ECG and PCG has been successfully applied to cardiovascular monitoring and disease classification (Li et al., 2025; Tran et al., 2025). Nevertheless, both signals represent indirect observations and cannot independently reconstruct the coupling between electrical activity and mechanical contraction (Sun et al., 2024). Thus, acquiring cEMC dynamic characteristics from ECG and PCG signals remains a significant challenge.

From a systems science perspective, cEMC can be characterized as a dynamical system that integrates electrical excitation with mechanical contraction. In this view, state-space modeling provides a natural framework for describing such systems by inferring latent-state trajectories from observed sequences (Koppe et al., 2019). Within this framework, ECG and PCG serve as noisy, partial observations of the system, whereas critical determinants, including conduction velocity, calcium transients, and ventricular pressure, constitute latent states that are not directly measurable and must be inferred (Chen et al., 2010b). However, classical state-space models typically rely on predefined transition functions with fixed parameters, which may be inadequate for describing the high-order and time-varying dynamics of cEMC during incremental exercise (Antonacci et al., 2023, 2025). Consequently, reconstructing the dynamic structure of cEMC from limited observations without explicit physiological constraints is necessary.

Takens’ delay embedding theorem provides a theoretical foundation for reconstructing latent dynamical structures from observed signals. For physiological systems governed by deterministic nonlinear dynamics, delay-coordinate embedding has demonstrated that a phase space equivalent to the original system can be reconstructed from time-delayed observations of the measured variables (Gottwald and Reich, 2021). Although physiological systems are inevitably influenced by noise, perturbations, and regulatory variability, ECG and PCG signals may still preserve essential dynamical information about cardiac electromechanical activity, which can approximate the latent phase-space trajectory of cEMC through delay-coordinate reconstruction (Zia et al., 2021). Therefore, integrating Takens’ delay embedding with state-transition models offers a potential approach to characterizing cEMC dynamics using non-invasive physiological measurements. However, traditional linear state-transition models, such as vector autoregression (VAR), typically assume time-invariant coefficient matrices, which may not adequately describe the continuously adapting nature of cardiovascular regulation during incremental exercise (Antonacci et al., 2023). Therefore, it is necessary to utilize parameter estimation approaches that enable the matrix coefficients to evolve over time to capture the dynamic characteristics of cEMC. Recent advances in artificial intelligence techniques have enabled the modeling of time-varying dynamical systems using data-driven approaches (Yu and Wang, 2024). Neural network-based methods estimate nonlinear state-transition functions through flexible function approximation, providing powerful representations of complex temporal dynamics (Chen et al., 2010a). However, such approaches generally focus on predictive performance and provide limited insight into the dynamic structure among physiological subsystems. By contrast, graph-based modelling offers a structurally interpretable framework by representing interacting variables as nodes and their time-varying dependencies as evolving edges, thereby enabling the direct characterization of dynamic coupling patterns (Garcia-Retortillo and Ch Ivanov, 2025). Nonetheless, dynamic graph approaches have not yet been explored for modelling cardiac electromechanical coupling during exercise.

Building on these considerations, this study attempts to investigate a data-driven approach to extract the latent dynamical characteristics of cEMC from limited observations during incremental exercise. First, considering the physiological characteristics of cEMC during incremental exercise, the Takens delayed embedding method is used to reconstruct the phase space of the ECG and PCG. Then, the joint phase-space dynamics of the ECG and PCG are treated as state variables, and a VAR model is used to characterize local dynamics as a locally linear approximation of the nonlinear system. Given the time-varying nature of cEMC during exercise, a time-varying parameter estimation strategy is incorporated, informed by dynamic graph-based modeling. The findings are expected to facilitate the extraction of dynamical features characterizing cEMC responses to incremental exercise, thereby providing a methodological basis for assessing exercise-related cardiovascular risk.

## 2 Materials and methods

### 2.1 Datasets

This study used the EPHNOGRAM dataset to evaluate the effectiveness of the proposed analysis approach (Kazemnejad et al., 2024). This dataset includes synchronized ECG and PCG data from 24 healthy subjects (age: 25.4 ± 1.9 years) during rest, bicycle ergometer testing, and Bruce protocol treadmill incremental exercise testing. All signals were simultaneously recorded using a three-lead ECG device and a single PCG stethoscope at a sampling frequency of 8000 Hz. This dataset was approved by Shiraz University’s Biomedical Engineering Review Board. Since the ECG and PCG signals from the Bruce protocol treadmill stress test contained numerous artifacts and exhibited poor data quality, this study selected only data from bicycle ergometer tests of 10 subjects for subsequent analysis, with each subject contributing a 30-minute recording.

Previous studies have demonstrated that by extracting the R-peak to R-peak interval (RRI) from a 2-minute ECG segment and applying detrended fluctuation analysis (DFA), the DFA α_1_ index can be obtained and used to evaluate exercise intensity (Gronwald et al., 2020). Specifically, during low-, moderate-, and high-intensity exercise, the DFA α_1_ values are approximately 1.0, 0.75, and ≤0.5, respectively (Gronwald et al., 2020). Therefore, this study stratified DFA α_1_ into four grades (Zone 0, Zone 1, Zone 2, Zone 3) using the aforementioned critical thresholds.

### 2.2 Signal processing

Given the significant differences in the physical characteristics of ECG and PCG signals, and the widespread presence of noise, including baseline drift, electromyographic interference, and motion artifacts, in the raw signals, the signals were preprocessed before modelling. The preprocessing procedures primarily include sliding-window segmentation, signal denoising, envelope extraction, outlier cleaning, extraction of cardiac electromechanical intervals, and DFA analysis. The detail can be found in the **Supplementary File**.

### 2.3 Phase space reconstruction

Since raw ECG and PCG recordings, as one-dimensional scalar time series, do not fully represent the nonlinear dynamics of the cardiac dynamical system, the recordings are resampled to 1000 Hz and their phase spaces are reconstructed using Takens’ delay-embedding theorem, resulting in state vectors $X(t)={\left[x(t),\;x(t+\tau ),\;\ldots ,\;x(t+(m-1)\tau )\right]}^{⊤}$. The delay time *τ* is determined by the first local minimum of the mutual information function, thereby minimizing redundancy across embedding dimensions (Fraser and Swinney, 1986). The embedding dimension *m* is selected using the false nearest neighbor (FNN) algorithm as the smallest *m* for which the FNN ratio falls below 1% (Kennel et al., 1992). Optimal values of *τ* ( $\tau_{ecg,\text{ best}}=103.2\;\pm \;4.7,\;\tau_{pcg,\text{ best}}=121.5\;\pm \;1.6$) and *m* ( $m_{ecg,\text{ best}}=6.2\;\pm \;0.1,\;m_{pcg,\text{ best}}=5.9\;\pm \;0.1$) for each signal are initially identified through a grid search across all segments. Finally, the individual ECG and PCG state vectors are concatenated to form a single *n_s_*-dimensional (*n_s_* is the sum of *m_ecg_* and *m_peg_*) joint state vector *s_k_* for subsequent modeling analysis.

### 2.4 Model architectures

The VAR and dynamic graph were integrated to construct a dynamic graph-based VAR (DG-VAR) model for dynamic analysis of cEMC. The effectiveness of this modeling approach was assessed through a comparative analysis of the DG-VAR model, traditional VAR models, and the time-varying extension (TV-VAR).

#### 2.4.1 VAR

The dynamic model of the joint state vector *s_k_* was constructed using the VAR. The mathematical expression of the model is as shown in Equation 1:

(1) $$s_{k}=\Phi s_{k-1}+\epsilon_{k},\;\;\;\epsilon_{k}∽N(0,\;\Sigma )$$

Where $\Phi \in R^{n_{s}\times n_{s}}$is the time-invariant coefficient matrix, held constant within each sliding window. *ϵ_k_* represents independently and identically distributed random noise terms. Takens embedding supplies a locally linear, noise-projected representation of the cardiac dynamics, but it does not render the observed series weakly stationary. Since the VAR coefficient estimation requires weak stationarity to avoid spurious regression bias, this study applies differencing operations to the input dataset. To distinguish the self-evolution patterns of ECG and PCG as well as their coupling relationship, the coefficient matrix Φ is decomposed into blocks as shown in the Equation 2

(2) $$\Phi =[\begin{array}{cc} {\text{A}}_{\text{yy}} & {\text{A}}_{\text{yx}}\\ {\text{A}}_{\text{xy}} & {\text{A}}_{\text{xx}} \end{array}]$$

where ${\text{A}}_{\text{yy}}\in {\text{R}}^{{\text{m}}_{\text{pcg}}\times {\text{m}}_{\text{pcg}}}$and ${\text{A}}_{\text{xx}}\in {\text{R}}^{{\text{m}}_{\text{ecg}}\times {\text{m}}_{\text{ecg}}}$ describe the autoregressive inertia of PCG and ECG. ${\text{A}}_{\text{yx}}\in {\text{R}}^{{\text{m}}_{\text{pcg}}\times {\text{m}}_{\text{ecg}}}$ denotes the electromechanical forward pathway by which cardiac electrical activity influences the subsequent contraction of the myocardium. In contrast, ${\text{A}}_{\text{xy}}\in {\text{R}}^{{\text{m}}_{\text{ecg}}\times {\text{m}}_{\text{pcg}}}$ corresponds to the feedback pathway in which myocardial mechanical contraction affects cardiac electrical activity. Given the state sequence of the training set be $\left\{{\text{S}}_{0},\;{\text{S}}_{1},\;\ldots ,\;{\text{S}}_{T-1}\right\}$, the lag and the target matrices are as shown in Equation 3:

(3) $$S_{prev}=[\begin{array}{c} S_{0}^{⊤}\\ \vdots \\ S_{T-2}^{⊤} \end{array}]\in {\text{R}}^{(T-1)\times n_{s}},\;\;\;\;\;S_{target}=[\begin{array}{c} S_{1}^{⊤}\\ \vdots \\ S_{T-1}^{⊤} \end{array}]\in {\text{R}}^{(T-1)\times n_{s}}$$

The coefficient matrix Φ was estimated using ridge regression, which avoids singularity and suppresses overfitting. The loss function is as shown in Equations 4-6:

(4) $$L_{\text{rec}}=∥S_{t\arg et}\;-S_{prev}\Phi^{⊤}{∥}_{\text{F}}^{2}$$

(5) $$L_{\text{reg}}=∥\Phi {∥}_{\text{F}}^{2}$$

(6) $$L\;=\;L_{\text{rec}}+{\text{λ}}_{1}L_{\text{reg}}$$

Where $L_{\text{rec}}$ denoted the reconstruction term and $L_{\text{reg}}$ denoted the L2 regulaization. The L2 regularization coefficient ${\text{λ}}_{1}$was set to 10–^4^ as determined by cross validation, which corresponds to the optimal intersection point of the prediction error plateau and the dynamic stability.

#### 2.4.2 TV-VAR

This study extends the VAR model by incorporating time-varying parameter estimation. Specifically, the coefficient matrix Φ in the VAR model is replaced with a sequence of time-varying matrices $\left\{\Phi_{k}\right\}$, while the disturbance covariance matrix is also allowed to be time-varying. The mathematical expression of the model is shown in Equation 7:

(7) $$s_{k}=\Phi_{k}s_{k-1}+\epsilon_{k},\;\;\;\;\;\epsilon_{k}∽N(0,\;\Sigma_{k})$$

Both $\Phi_{k}\in {\text{R}}^{n_{s}\times n_{s}}$ and $\Sigma_{k}\in {\text{R}}^{n_{s}\times n_{s}}$ are generated using a two-layer fully connected neural network with a local history window of length 5 and the hyperbolic tangent activation function. Meanwhile, a time-varying smoothness regularization term is embedded into the loss function to penalize abrupt parameter variations, thereby ensuring the continuous evolution of cardiac dynamic. The loss function is as shown in the Equation 8.

(8) $$L=L_{\text{rec}}+{\text{λ}}_{1}L_{\text{reg}}+{\text{λ}}_{2}L_{\text{smooth}}$$

where ${\text{λ}}_{1}$ is the L2 regularization coefficient and $\;{\text{λ}}_{2}$ is the time-varying smoothness regularization coefficient, both set to 10^-4^. The network is trained using the Adam optimizer with a learning rate of 10–^2^ for 100 epochs.

#### 2.4.3 DG-VAR

Since the TV-VAR model employs a fully dense coefficient matrix that assumes default coupling among all state nodes, it fails to distinguish genuine physiological causal connections from noise-induced spurious correlations (Mody and Rangarajan, 2022). To address this limitation, this study introduces a range constraint and L1 sparsity regularization to transform the coefficient matrix into the adjacency matrix of a dynamic graph, thereby enabling the automatic selection of valid causal pathways (Wehmuth et al., 2015). The mathematical expression of the model is as shown in Equations 9 and Equation 10:

(9) $$s_{k}=A_{k}s_{k-1}+\epsilon_{k},\;\;\;\;\;\epsilon_{k}∽N(0,\;\Sigma_{k})$$

(10) $$A_{k}=\tanh(W_{adj}h_{k}+b_{adj}),\;\;|A_{k},ij|<1$$

Where *A_k_* denotes the time-varying adjacency matrix at time *k*, *W_adj_* and *b_adj_* represent the output-layer parameters, and h*_k_* is the hidden-layer feature representation. The hyperbolic tangent activation constrains matrix elements to [−1,1], where positive, negative, and zero values indicate promoting, inhibitory, and absent connections, respectively. An additional L1 regularization term is incorporated into the loss function to promote sparse coupling patterns by shrinking insignificant connections toward zero. The loss function is shown in Equation 11.

(11) $$L=L_{\text{rec}}+{\text{λ}}_{1}L_{\text{reg}}+{\text{λ}}_{2}L_{\text{smooth}}+{\text{λ}}_{\text{g}}L_{\text{sparse}}$$

Where ${\text{λ}}_{\text{g}}$ is the graph sparsity regularization coefficient (In this study, ${\text{λ}}_{\text{g}}$ was set to 0.01). The $L_{\text{sparse}}$ is the L1 sparsity regularization term, which drives the weights of ineffective couplings toward zero by computing the mean of the L1 norms of the adjacency matrices *A_k_* over all time steps. The calculation equation for $L_{\text{sparse}}$ is shown in Equation 12.

(12) $$L_{\text{sparse}}=\frac{1}{T^{\prime }}\sum_{k=1}^{T^{\prime }}∥A_{k}{∥}_{1}$$

In Equation 12, the L1 regularization is equivalent to imposing a Laplace prior on the adjacency matrix, enabling automatic selection of sparse causal pathways.

### 2.5 Model evaluation

Each signal segment was split chronologically into training and test sets, with the initial 80% allocated to training and the remaining 20% retained for testing. The root mean square error (RMSE) and mean absolute percentage error (MAPE) were subsequently computed on test-set samples to quantitatively evaluate the model’s predictive performance.

### 2.6 Dynamics characteristics

The cEMC dynamics were characterized from three perspectives: stationarity, coupling strength, and dynamic graph topology.

#### 2.6.1 Stationarity indicators

Stationarity was assessed by calculating the spectral radius of the coefficient submatrix (Shojaie and Fox, 2022). The spectral radius of any given submatrix A is defined in Equation 13:

(13) $$\rho (\text{A})=max_{i}|\lambda_{i}(\text{A})|,\;\text{A}\in \{A_{xx},\;A_{yy},\;A_{xy},\;A_{yx}\}$$

where $\lambda_{i}(\text{A})$ denotes the i-th eigenvalue of submatrix A. Temporal variations in subsystem stability were evaluated by the average of the spectral radius sequence. The subsystem spectral radius was independently calculated for both ECG and PCG to characterize their intrinsic autoregressive dynamics. A spectral radius approaching 1 indicates a stronger autoregressive effect, while values exceeding 1 suggest potential local divergence.

#### 2.6.2 Coupling strength indicators

Metrics were derived from the cross-channel coefficient submatrices ( $A_{xy}\;\text{and}\;A_{yx}$) to quantify the strength of directional coupling. First, the average coupling magnitude was calculated as the mean absolute value of all matrix elements, representing the overall interaction intensity (Faes et al., 2012). Second, the cross-channel synergistic strength was defined as the mean absolute value of the off-diagonal coefficients, which specifically reflects information exchange between different signal dimensions (Schiatti et al., 2015). Taking $A_{yx}$ as an example, the average coupling magnitude, and cross-channel synergistic strength are calculated using Equations 14, 15.

(14) $$\bar{\left|A_{yx}\right|}=\frac{1}{n_{y}n_{x}}\sum_{i=1}^{n_{y}}\sum_{j=1}^{n_{x}}|A_{yx},ij|$$

(15) $$Synergy(A_{yx})=\frac{1}{\left|O\right|}\sum_{(i,j)\in O}|A_{yx},ij|$$

Where |*O*| denotes the cardinality of set *O*, corresponding to the total count of off-diagonal entries in the matrix. The index set O={(i,j):i≠j} collects all ordered row-column index pairs associated with the matrix’s off-diagonal elements. The bidirectional coupling strength was determined by integrating both forward and feedback coupling pathways. It is defined as Equation 16:

(16) $$E=∥A_{yx}{∥}_{F}+∥A_{xy}{∥}_{F}$$

Where $∥\cdot {∥}_{F}$ is the Frobenius norm. *E* is the bidirectional coupling energy.

#### 2.6.3 Dynamic graph topology indicators

Based on the sparse causal graph adjacency matrix sequence $\left\{A_{k}\right\}$, graph sparsity, average edge strength, and graph evolution smoothness were extracted. Graph sparsity was defined as the proportion of weak connections below a predefined threshold ( θ=0.05), reflecting the structural sparsity of the causal network (Mody and Rangarajan, 2022). Average edge strength quantified the global interaction intensity among state variables (Marzbanrad et al., 2020). Graph evolution smoothness measured temporal variations in network topology and coupling weights between adjacent time points, with lower values indicating more stable network evolution (Barnett and Seth, 2015). The above dynamic graph topology metrics were calculated using Equations 17–19.

(17) $$\text{Graph Sparsity}=\frac{1}{\tau^{\prime }}\sum_{k}\frac{\#\{(i,j):\;|A_{k,\;ij}|<\theta \}}{n_{s}^{2}}$$

(18) $$\text{Graph Edge}=\frac{1}{\tau^{\prime }}\sum_{k}\frac{1}{n_{s}^{2}}\sum_{i,j}|A_{k,\;ij}|$$

(19) $$\text{Graph Smooth}=\frac{1}{T'-1}\sum_{k}∥A_{k+1}-A_{k}{∥}_{F}$$

Where *T’* is the total number of effective time windows. *n_s_* is the dimension of the joint state vector. $A_{k,\;ij}$ is the coupling weight in the i-th row and j-th column of the causal matrix at time k, and θ is the weak connection threshold. $∥\cdot {∥}_{F}$ denotes the Frobenius norm, and #{·} is the number of elements satisfying the condition.

### 2.7 Statistical analysis

In this study, continuous data were described statistically using the median and the 25th and 75th percentiles. Welch’s one-way analysis of variance (ANOVA) was used to analyze differences in model parameters across different exercise intensity levels. Generalized mixed-effects regression was performed to analysis model parameters associated with cEMC parameters. All statistical computations were conducted via Jamovi software (version 1.8.1). A two-tailed *p*-value < 0.05 was considered statistically significant for all tests.

## 3 Results

This study demonstrates that the DG-VAR model exhibits significantly superior fitting and prediction performance for the ECG and PCG phase spaces than the VAR and TV-VAR models. The error analysis shows that in ECG phase-space forecasting, DG-VAR achieves a 58.8% reduction in median RMSE and a 60.5% reduction in median MAPE compared with VAR, and a 17.0% reduction in median RMSE and a 23.6% reduction in median MAPE relative to TV-VAR. Similarly, DG-VAR reduces the median RMSE and median MAPE for PCG prediction by 74.2% and 74.8% relative to VAR, and by 37.5% and 43.0% relative to TV-VAR. Further analysis stratified by exercise intensity shows that DG-VAR yields the lowest error in predicting both ECG and PCG signals, and its accuracy remains robust across all exercise intensity zones (**Table 1**). Although DG-VAR exhibits elevated errors for both signals in Zone 3, these errors are still smaller than those of VAR and TV-VAR. This finding suggests that DG-VAR exhibits robust performance advantages across different exercise intensities.

**Table 1:** The predictive performance of different models in estimating ECG and PCG.

Signal	Exercise intensity	VAR		TV-BAR		DG-VAR	
		RMSE	MAPE	RMSE	MAPE	RMSE	MAPE
ECG	Overall	0.0452 (0.0355, 0.0532)	19.67 (14.50, 27.01)	0.0224 (0.0145, 0.0289)	10.16 (6.67, 14.35)	0.0186 (0.0096, 0.0252)	7.76 (4.63, 11.69)
	Zone 0	0.0452 (0.0357, 0.0535)	18.77 (14.46, 26.59)	0.0225 (0.0151, 0.0289)	10.12 (6.70, 14.05)	0.0187 (0.0106, 0.0254)	7.81 (4.83, 11.19)
	Zone 1	0.0356 (0.0251, 0.0469)	18.07 (12.96, 26.30)	0.0146 (0.0092, 0.0207)	7.19 (4.15, 12.43)	0.0087 (0.0036, 0.0190)	3.38 (1.66, 8.89)
	Zone 2	0.0517 (0.0427, 0.0523)	20.73 (17.71, 25.69)	0.0273 (0.0192, 0.0303)	10.16 (7.41, 15.73)	0.0236 (0.0129, 0.0265)	7.56 (4.57, 12.89)
	Zone 3	0.0478 (0.0436, 0.0553)	29.28 (20.85, 35.15)	0.0264 (0.0234, 0.0325)	17.93 (12.47, 20.60)	0.0197 (0.0170, 0.0282)	15.00 (8.09, 18.13)
PCG	Overall	0.0329 (0.0267, 0.0417)	17.65 (13.23, 22.43)	0.0136 (0.0112, 0.0185)	7.81 (5.68, 9.86)	0.0085 (0.0059, 0.0116)	4.45 (2.69, 6.27)
	Zone 0	0.0312 (0.0260, 0.0397)	17.61 (12.88, 22.83)	0.0132 (0.0109, 0.0178)	7.77 (5.49, 9.72)	0.008 (0.0058, 0.0110)	4.21 (2.52, 6.22)
	Zone 1	0.0395 (0.0375, 0.0442)	16.64 (14.60, 19.60)	0.0169 (0.0155, 0.0191)	6.78 (6.44, 9.47)	0.0114 (0.0094, 0.0128)	4.15 (4.03, 6.10)
	Zone 2	0.0389 (0.0325, 0.0418)	20.42 (13.90, 21.13)	0.0166 (0.0147, 0.0193)	7.85 (7.03, 10.73)	0.0095 (0.009, 0.0122)	4.77 (4.06, 6.43)
	Zone 3	0.0395 (0.0348, 0.0500)	17.92 (16.33, 23.58)	0.0187 (0.0155, 0.0248)	9.47 (9.02, 13.05)	0.0115 (0.0085, 0.0141)	4.97 (4.56, 6.74)

Welch’s one-way ANOVA identified significant differences among exercise intensity levels for all matrix-derived parameters (all *p* < 0.001). The intra-individual variations of the model-derived parameters are presented on the secondary axis of **Figure 1**. The spectral radius of A*_xx_* demonstrates a J-shaped increase with exercise intensity, while the spectral radius of A*_yy_* follows an inverse pattern. Forward coupling submatrix parameters, including the spectral radius, average magnitude, and synergistic strength of A*_yz_*, increase in a consistent manner with exercise intensity. In contrast, the spectral radius of the feedback coupling submatrix exhibits a logarithmic growth curve trend. The bidirectional coupling energy increases monotonically across exercise intensity zones. Graph sparsity exhibits an initial increase followed by a decrease as exercise intensity. The average edge weight of dynamic graphs follows a J-shaped growth trajectory, whereas smoothness displays an exponential growth trend. The relationship between the model-derived and cEMC parameters as shown in **Table 2**.

**Figure 1:**
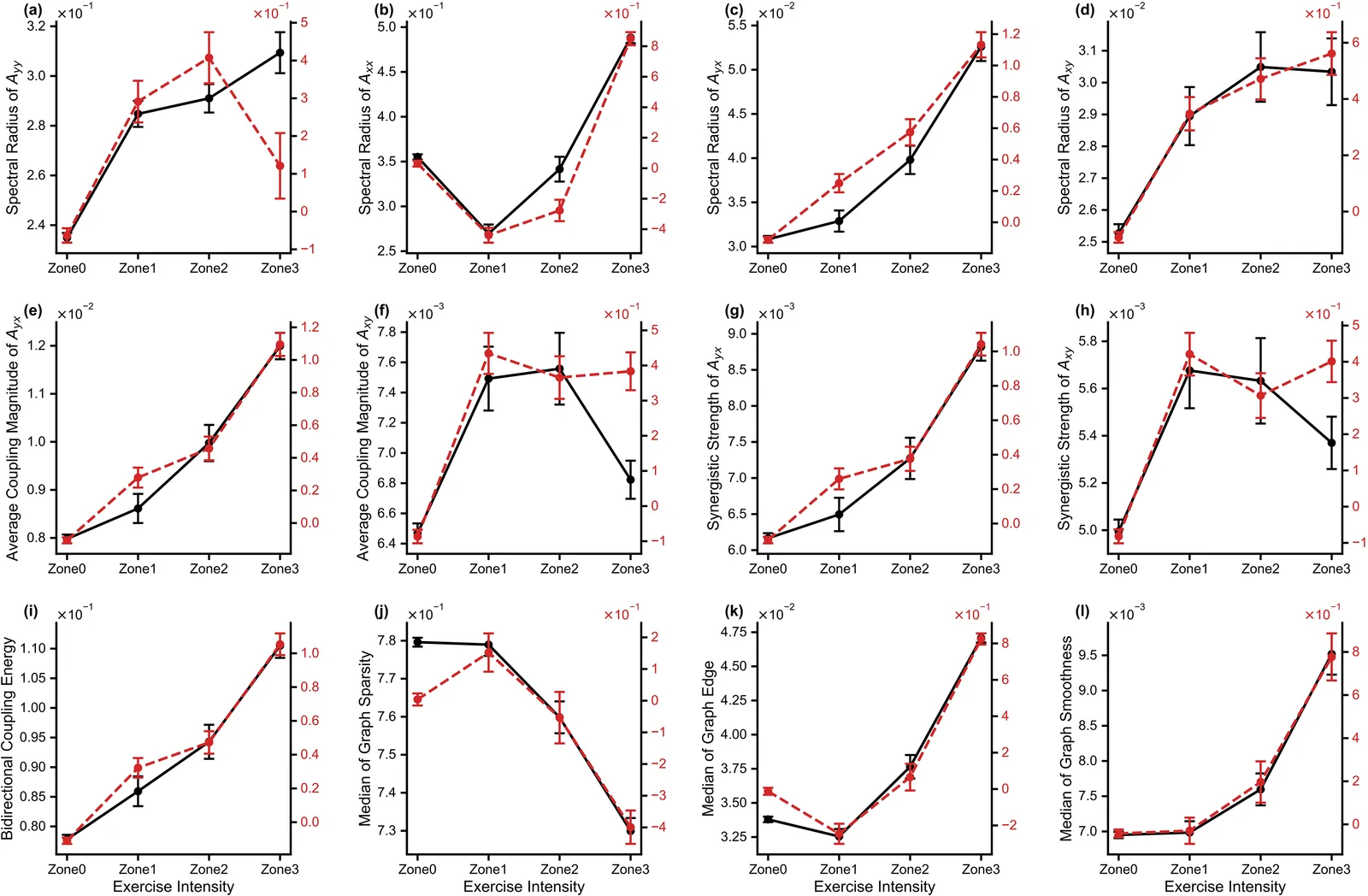
The distributions of model-derived parameters across exercise intensity zones. **(a)** spectral radius of A*_yy_*; **(b)** spectral radius of A*_xx_*; **(c)** spectral radius of A*_yx_*; **(d)** spectral radius of A*_xy_*; **(e)** average coupling magnitude of A*_yx_*; **(f)** average coupling magnitude of A*_xy_*; **(g)** synergistic strength of A*_yx_*; **(h)** synergistic strength of A*_xy_*; **(i)** bidirectional coupling energy; **(j)** median of graph sparsity; **(k)** median of graph edge; **(l)** median of graph smoothness. The primary axis is the raw value, and the secondary axis is the intra-individual Z-score.

**Table 2:** The generalized mixed-effects regression results between model-derived parameters and cEMC parameters.

Predictors	Q-S1 interval		Q-S2 interval		S1-S2 interval	
	*β* (95% CI)	*p*-value	*β* (95% CI)	*p*-value	*β* (95% CI)	*p*-value
Spectral Radius of A*_yy_*	0.112 (0.042, 0.183)	0.002	0.574 (0.509, 0.639)	<0.001	0.706 (0.640, 0.773)	<0.001
Spectral Radius of A*_xx_*	0.600 (0.488, 0.712)	<0.001	1.597 (1.492, 1.701)	<0.001	1.779 (1.673, 1.886)	<0.001
Spectral Radius of A*_yz_*	-0.254 (-0.376, -0.131)	<0.001	-0.075 (-0.189, 0.039)	0.196	0.074 (-0.042, 0.191)	0.212
Spectral Radius of A*_xy_*	-0.095 (-0.207, 0.017)	0.098	0.093 (-0.011, 0.197)	0.080	0.096 (-0.010, 0.203)	0.077
Average Coupling Magnitude of A*_yz_*	0.060 (-0.171, 0.291)	0.612	0.004 (-0.210, 0.218)	0.971	-0.128 (-0.347, 0.091)	0.252
Average Coupling Magnitude of A*_xy_*	-0.487 (-0.697, -0.276)	<0.001	0.102 (-0.094, 0.297)	0.307	0.557 (0.358, 0.757)	<0.001
Synergistic Strength of A*_yz_*	0.341 (0.166, 0.516)	<0.001	0.609 (0.446, 0.771)	<0.001	0.629 (0.463, 0.795)	<0.001
Synergistic Strength of A*_xy_*	0.759 (0.602, 0.915)	<0.001	0.394 (0.248, 0.539)	<0.001	0.002 (-0.146, 0.151)	0.975
Bidirectional Coupling Energy	-0.844 (-1.017, -0.670)	<0.001	-1.742 (-1.903, -1.581)	<0.001	-1.807 (-1.972, -1.643)	<0.001
Median of Graph Sparsity	-0.302 (-0.356, -0.248)	<0.001	-0.394 (-0.444, -0.344)	<0.001	-0.313 (-0.363, -0.262)	<0.001
Median of Graph Edge	-0.910 (-1.036, -0.784)	<0.001	-1.805 (-1.922, -1.688)	<0.001	-1.853 (-1.973, -1.734)	<0.001
Median of Graph Smoothness	0.197 (0.165, 0.230)	<0.001	0.089 (0.059, 0.119)	<0.001	0.004 (-0.027, 0.034)	0.809

## 4 Discussion

In state-space modeling, system dynamics are represented by a coefficient matrix that functions as a transition operator, describing the evolution of system states over time (Hall et al., 2019). VAR, as a simplified variant of these models, is widely used for physiological signal analysis. However, conventional VAR assumes a constant transition operator, resulting in a stationary linear mapping of system interactions (Madhav et al., 2024). This assumption may not adequately represent cEMC during exercise, in which autonomic regulation, hemodynamic responses, and myocardial contractility continuously adapt to increasing physiological demands (Wan et al., 2023). Consequently, the coupling dynamics are expected to evolve, requiring temporal adaptation of the linear operator. Time-varying coefficient matrices allow the operator to adapt dynamically (Kostoglou et al., 2019). However, without structural constraints, temporal variation in the coefficient matrix introduces excessive degrees of freedom, increasing the risk of fitting non-physiological fluctuations as coupling alterations, especially in the presence of motion artefacts or measurement noise, which can compromise model generalizability. Dynamic graph modeling provides a potential solution to address these limitations by introducing structural constraints into the evolution of the coefficient matrix. Specifically, the distribution of non-zero coefficients is modeled as a time-varying network topology, representing the changing organization of effective interactions among system states (Mei et al., 2025). Instead of assuming full connectivity, this approach assumes that only a limited subset of interactions contributes to system evolution at each time point. Such sparsity constraints reduce the effective dimensionality of the parameter space and restrict parameter estimation to informative connections, thereby mitigating the influence of non-physiological fluctuations (Abbas et al., 2025). Following this principle, the present study integrates dynamic graph topology constraints into time-varying VAR structures to develop DG-VAR. Error analysis demonstrates the effectiveness of this modeling approach, showing that the model simultaneously supports adaptive temporal coupling evolution and topology-driven sparse regularization, thereby overcoming the dual limitations of time-invariance and lack of structural constraints inherent in traditional fixed VAR transfer operators.

Rather than treating cardiac electrical and mechanical activity as independent processes, DG-VAR characterizes cEMC as an evolving bidirectional interaction within their joint phase space, with the time-varying cross-block coefficients capturing the dynamic influences from electrical to mechanical activity and vice versa. In this study, the matrix-derived parameters of DG-VAR exhibited significant correlations with the cardiac electromechanical interval, suggesting their ability to reflect exercise-induced variations in cEMC. From a dynamical systems perspective, these parameters describe cEMC adaptation, including the persistence of system-state evolution, the strength of electromechanical interactions, and the adaptive organization of cEMC network dynamics. The spectral radius characterizes the persistence of system-state evolution and reflects the dynamic stability of cEMC in response to exercise intensity (Christini et al., 1995). Decomposition of the coefficient matrix revealed distinct adaptive patterns among functional pathways. The spectral radius of the electromechanical forward coupling submatrix increased progressively with exercise intensity, which suggests that transmission from electrical activation to mechanical contraction was enhanced during periods of increased cardiac demand (Tibbits et al., 1981). Conversely, the spectral radius of the mechanoelectrical feedback coupling submatrix appears to exhibit a saturation effect. Although feedback modulation increases within the range of low to moderate exercise intensity, the coupling strength does not increase correspondingly under higher-intensity stimulation (Hatano et al., 2025). Beyond the state stability, the coupling strength and graph topology revealed the progressive reorganization of cEMC interactions. Increased bidirectional coupling energy reflected enhanced information exchange between electrical and mechanical activity, which was further supported by their inverse associations with the Q-S1, Q-S2, and S1-S2 intervals. Meanwhile, graph-based analysis demonstrated decreased network sparsity and increased edge strength with increasing exercise intensity, suggesting a transition toward a more densely interconnected cEMC. The exponential increase in the smoothness of graph evolution further suggested enhanced temporal organization of cEMC under elevated physiological demands (Garcia-Retortillo and Ch Ivanov, 2025). Collectively, these findings suggest that exercise-induced cEMC adaptation emerges from coordinated adjustments in state persistence, electromechanical interactions, and adaptive organization of network dynamics, rather than isolated alterations in individual cardiac components.

Despite these findings being promising, several limitations should be acknowledged. Firstly, the identified cEMC characteristics are mathematical latent features derived from computational models rather than direct physiological measurements. The EPHNOGRAM dataset lacks relevant echocardiographic measures, such as electromechanical intervals and ventricular mechanical synchrony, precluding direct validation against myocardial electrical activation and mechanical contraction. Therefore, the physiological significance of these model-derived features requires validation using independent physiological measurements in future studies. Secondly, the limited sample size and population homogeneity may restrict the generalizability of the findings. The EPHNOGRAM dataset exclusively comprises young, healthy male individuals, and further research is necessary to ascertain whether the identified cEMC dynamics are consistent across populations. Thirdly, the evaluation of exercise intensity was constrained by the lack of metabolic measurements. While DFA α_1_ reflects non-linear autonomic dynamics associated with exercise stress, it does not directly correspond to metabolic load as measured by VO_2_, which is considered the gold standard for assessing exercise intensity (Meyer et al., 2005). The absence of VO_2_ data in the EPHNOGRAM dataset precludes independent calibration of exercise intensity based on metabolic demand. Future research could validate the relationship between exercise intensity and cEMC dynamic characteristics further by incorporating metabolic load measures. Finally, the selection of delay estimation strategy can affect the dynamics of the reconstructed phase space, which is a methodological limitation of this study. The sensitivity of cEMC dynamic reconstruction to different time delay estimation strategies is an important question for future research.

## 5 Conclusion

In summary, the DG-VAR model proposed in this study achieves outstanding performance in predicting the joint phase space of ECG and PCG signals during incremental exercise. Across all exercise intensity levels, the model consistently achieves superior prediction accuracy and robustness compared to conventional VAR and its time-varying extension. Analysis of matrix-derived parameters reveals an intensity-dependent regulatory pattern in cardiac electromechanical dynamics: the forward electromechanical coupling increases monotonically, the feedback mechanism exhibits a logarithmic growth curve trajectory, and the network dynamics topology progressively evolves toward denser and smoother configurations. These dynamic characteristics show significant correlations with the Q-S1, Q-S2, and S1-S2 intervals. This non-invasive, label-free, data-driven analytical framework offers a novel, physiologically interpretable approach to dynamic monitoring of exercise-induced cEMC responses.

## Data Availability

Publicly available datasets were analyzed in this study. This data can be found here: https://physionet.org/content/ephnogram/1.0.0/.
