# Supplementary material for: Dynamic graph vector autoregression for characterizing cardiac electromechanical dynamics during incremental exercise from the joint phase space of electrocardiogram and phonocardiogram
Source: Front Physiol. 2026 Sep 29;17:1946312. doi: 10.3389/fphys.2026.1946312 (PMC13657968; doi:10.3389/fphys.2026.1946312)
Supplement: Supplementary file 1 [file DataSheet1.pdf]

Given the significant differences in the physical characteristics of ECG and PCG signals, and the widespread presence of noise, including baseline drift, electromyographic interference, and motion artifacts, in the raw signals, the signals were preprocessed before modelling. The preprocessing procedures primarily include sliding-window segmentation, signal denoising, envelope extraction, outlier cleaning, extraction of cardiac electromechanical intervals, and DFA analysis.

(1) Sliding Window Segmentation: The ECG and PCG signals were segmented using a sliding-window approach under two distinct schemes. For model fitting and performance evaluation, non-overlapping 120-s segments were used to minimize the potential for information leakage between adjacent segments and thereby avoid overly optimistic estimates of model performance. In contrast, for feature interpretation, an overlapping segmentation strategy was adopted in accordance with the HRV-based exercise-intensity detection method proposed by (Gronwald et al., 2020). Specifically, the continuous signals were segmented using a 120-s sliding window with a 5-s step size. This overlapping scheme was chosen to provide higher temporal resolution for tracking transient physiological changes, particularly those occurring around abrupt transitions in exercise intensity, which may be inadequately captured by non-overlapping segmentation.

(2) ECG Signal Denoising: In this study, a fourth-order Butterworth band-pass filter with a passband of 0.5-4.0 Hz was implemented to eliminate motion artifacts and high-frequency noise components contained in raw ECG signals. Then, the Pan-Tompkins algorithm was used to detect R peaks in ECG signals (Pan and Tompkins, 1985). The RRI sequence was extracted based on the annotations of the detected R-wave peaks.

(3) PCG Signal Envelope Extraction: Raw PCG signals exhibit inherent high-frequency oscillatory characteristics, making the waveform hardly reflect the features of the first (S1) and second heart sound (S2). This study applied an absolute value operation to the raw PCG signal to obtain a single-sided amplitude signal. The Hilbert transform was further used to construct the analytic signal and extract the instantaneous amplitude envelope of the PCG signal, and a second-order Butterworth low-pass filter with a cutoff frequency of 20 Hz was used to smooth the envelope.

(4) Standardization: This study calculates the mean and standard deviation within a sliding 120-second window for time-adaptive standardization.

(5) Outlier Cleaning: Since beat-to-beat temporal feature sequences are susceptible to various interfering factors, including motion artifacts, respiratory fluctuations, and signal detection errors, which can generate anomalous noise inconsistent with typical physiological dynamics, they degrade the robustness of subsequent time-series modelling. Therefore, this study used the Local Weighted Scatter Smoothing (LOWESS) method to detect and correct outliers across all beat-to-beat temporal feature sequences, thereby suppressing interference from abnormal samples and improving the quality of the feature sequences (Cleveland, 1979).

(6) Cardiac Electromechanical Intervals Extraction: In accordance with the procedures established by (Kazemnejad et al., 2024), the location of S1 was identified using the R-peak and T-peak annotations. The cardiac electromechanical intervals from the QRS wave start point to S1 and

S2 (Q-S1, Q-S2), as well as the interval from S1 to S2 (S1-S2), were extracted for each beat.

(7) Detrended Fluctuation Analysis (DFA): DFA was applied to extract correlations within continuous RR interval sequences across multiple time scales, yielding the scaling exponents α_1 and α_2. In the DFA, α_1 quantifies short-term fluctuation properties, while α_2 describes long-term fluctuation characteristics. In accordance with established DFA protocols, the time scales were divided into short-range (4 – 16 beats) and long-range (16 – 64 beats) regimes, corresponding to α_1 and α_2, respectively (Shaffer and Ginsberg, 2017).

2.1. Phase Space Reconstruction

Since ECG and PCG signals are one-dimensional scalar time series data which cannot fully represent the non-linear dynamics of the cardiac dynamical system, this study resamples the raw signals to 1000 Hz and then performs high-dimensional phase-space reconstruction of the one-dimensional time-series signals based on Takens' embedding theory to obtain state vectors (Takens, 2006). The details are as follows:

(1) Takens Embedding Theorem: For a one-dimensional scalar time series $x(t)$ observed in a deterministic dynamical system, the phase-space vector can be reconstructed using the equal-interval delay embedding method. The reconstructed phase-space vector is expressed as follows:

$$X(t)=[x(t), x(t+\tau), \ldots, x(t+(m-1)\tau)]^{\top} \tag{1}$$

Where τ is the delay time and m is the embedding dimension.

(2) Delay Time Selection: In this study, the optimal delay time was identified based on the principle of minimizing mutual information (Fraser and Swinney, 1986). The mutual information at delay time is defined as:

$$I(\tau)=\sum_{x, y} p(x, y; \tau) log \frac{p(x, y; \tau)}{p(x)p(y)} \tag{2}$$

The τ value corresponding to the first local minimum of $I(\tau)$ is selected to minimise statistical dependence across the dimensions of the embedding vector, thereby allowing the geometric structure of the reconstructed attractor to be fully revealed.

(3) Embedded Dimension Selection: The False Nearest Neighbors (FNN) algorithm was adopted to identify the optimal embedding dimension, enabling the unfolded reconstruction of attractors (Kennel et al., 1992). The principle of the FNN algorithm was described as follows. For any given time point in the m-dimensional phase space, its nearest neighbor is defined as a true nearest neighbor if it remains adjacent when mapped to the m+1-dimensional phase space. In contrast, neighbors that only appear adjacent due to insufficient phase space dimensions are regarded as false nearest neighbors. The equation for calculating the false nearest neighbor ratio is presented below:

$$FNN\_ratio(m)=\frac{1}{N}\sum_{i=1}^{N} 1\left(\frac{\|X^{(m+1)}(t_i)-X^{(m+1)}(t_{nn})\|}{\|X^{(m)}(t_i)-X^{(m)}(t_{nn})\|}>r_1\right) \tag{3}$$

In Equation (3), m denotes the current embedding dimension. $X^{(m)}(t_i)$ and $X^{(m)}(t_{nn})$ represent the i phase point and its nearest neighbour, respectively, in the m-dimensional phase space. r_1 is the false nearest neighbour discrimination threshold (r_1=10). When the FNN ratio is less than 1%, the corresponding m is the optimal embedding dimension.

In this study, the optimal values of τ and m for each signal segment were determined using a grid search method. Then, the phase-space state vectors of the ECG and PCG were reconstructed using the optimized results for all segments to facilitate comparisons between different signal segments.

(4) Joint State Vector: Following the phase space reconstruction, Takens embedding is performed on the ECG and PCG sequences to obtain the ECG state vector $x_k=R^{m_{ecg}}$ and the PCG state vector $y_k=R^{m_{pcg}}$. The two sequences are aligned using optimal delay to construct an n_s-dimensional (n_s is the sum of m_{ecg} and m_{pcg}) joint ECG-PCG state vector:

$$s_k=\begin{bmatrix} y_k \\ x_k \end{bmatrix} \in R^{n_s} \tag{4}$$

References

Cleveland, W.S. (1979). Robust locally weighted regression and smoothing scatterplots. Journal of the American statistical association. 74, 829-836. doi:

Fraser, A.M., and Swinney, H.L. (1986). Independent coordinates for strange attractors from mutual information. Phys Rev A Gen Phys. 33, 1134-1140. doi: 10.1103/physreva.33.1134

Gronwald, T., Rogers, B., and Hoos, O. (2020). Fractal Correlation Properties of Heart Rate Variability: A New Biomarker for Intensity Distribution in Endurance Exercise and Training Prescription? Frontiers in Physiology. 11, 550572. doi: 10.3389/fphys.2020.550572

Kazemnejad, A., Karimi, S., Gordany, P., Clifford, G.D., and Sameni, R. (2024). An open-access simultaneous electrocardiogram and phonocardiogram database. Physiological Measurement. 45. doi: 10.1088/1361-6579/ad43af

Kennel, M.B., Brown, R., and Abarbanel, H.D. (1992). Determining embedding dimension for phase-space reconstruction using a geometrical construction. Physical Review A. 45, 3403-3411. doi: 10.1103/physreva.45.3403

Pan, J., and Tompkins, W.J. (1985). A real-time QRS detection algorithm. IEEE Transactions on Biomedical Engineering. 32, 230-236. doi: 10.1109/tbme.1985.325532

Shaffer, F., and Ginsberg, J.P. (2017). An Overview of Heart Rate Variability Metrics and Norms. Frontiers in Public Health. 5, 258. doi: 10.3389/fpubh.2017.00258

Takens, F. (2006). "Detecting strange attractors in turbulence", in: Dynamical Systems and Turbulence, Warwick 1980: proceedings of a symposium held at the University of Warwick 1979/80: Springer, 366-381.
